# A survey by the European Society of Breast Imaging on radiologists’ preferences regarding quality assurance measures of image interpretation in screening and diagnostic mammography

**DOI:** 10.1007/s00330-023-09973-7

**Published:** 2023-07-22

**Authors:** Eleni Michalopoulou, Paola Clauser, Fiona J. Gilbert, Ruud M. Pijnappel, Ritse M. Mann, Pascal A.T. Baltzer, Yan Chen, Eva Maria Fallenberg

**Affiliations:** 1 University of Nottingham, School of Medicine, Clinical Sciences Building, City Hospital Campus, Hucknall Road, NG5 1PB Nottingham, UK; 2 Department of Biomedical Imaging and Image-guided Therapy, Allgemeines Krankenhaus, Medical University of Vienna, 1090 Vienna, Austria; 3 Department of Radiology, Clinical School, Cambridge Biomedical Research Centre, University of Cambridge, Cambridge, CB2 0QQ UK; 4 University Medical Centre Utrecht, Utrecht University, Heidelberglaan 100, 3584 Utrecht, CX The Netherlands; 5 Dutch Expert Centre for Screening, Wijchenseweg 101, 6538 Nijmegen, SW The Netherlands; 6 Department of Medical Imaging, Radboud University Medical Centre, 6525 Nijmegen, The Netherlands; 7 Department of Radiology, The Netherlands Cancer Institute, Amsterdam, The Netherlands; 8 Department of Diagnostic and Interventional Radiology, School of Medicine & Klinikum Rechts der Isar, Technical University of Munich, Ismaninger Str. 22, 81675 München, Germany

**Keywords:** Mammography, Survey and questionnaires, Radiologists, Workplace, Workload

## Abstract

**Objectives:**

Quality assurance (QA) of image interpretation plays a key role in screening and diagnostic mammography, maintaining minimum standards and supporting continuous improvement in interpreting images. However, the QA structure across Europe shows considerable variation. The European Society of Breast Imaging (EUSOBI) conducted a survey among the members to collect information on radiologists’ preferences regarding QA measures in mammography.

**Materials and methods:**

An anonymous online survey consisting of 25 questions was distributed to all EUSOBI members and national breast radiology bodies in Europe. The questions were designed to collect demographic characteristics, information on responders’ mammography workload and data about QA measures currently used in their country. Data was analysed using descriptive statistical analysis, the *χ*^2^ test, linear regression, and Durbin-Watson statistic test.

**Results:**

In total, 251 breast radiologists from 34 countries completed the survey. Most respondents were providing both screening and symptomatic services (137/251, 54.6%), working in an academic hospital (85/251, 33.9%) and reading 1000–4999 cases per year (109/251, 43.4%). More than half of them (133/251, 53%) had established QA measures in their workplace. Although less than one-third (71/251, 28.3%) had to participate in regular performance testing, the vast majority (190/251, 75.7%) agreed that a mandatory test would be helpful to improve their skills.

**Conclusion:**

QA measures were in place for more than half of the respondents working in screening and diagnostic mammography to evaluate their breast imaging performance. Although there were substantial differences between countries, the importance of having QA in the workplace and implemented was widely acknowledged by radiologists.

**Clinical relevance statement:**

Although several quality assurance (QA) measures of image interpretation are recommended by European bodies or national organisations, the QA in mammography is quite heterogenous between countries and reporting settings, and not always actively implemented across Europe.

**Key Points:**

- *The first survey that presents radiologists’ preferences regarding QA measures of image interpretation in mammography.*
- *Quality assurance measures in the workplace are better-established for breast screening compared to diagnostic mammography.*
- *Radiologists consider that performance tests would help to improve their mammography interpretation skills.*

**Supplementary Information:**

The online version contains supplementary material available at 10.1007/s00330-023-09973-7.

## Introduction

Breast cancer is the most common cancer among European women with an estimated number of 576,300 patients diagnosed in 2020 [1]. It is estimated that approximately 1 in 11 females will be diagnosed with invasive breast cancer at some point during their lifetime [1].

Regular mammography screening is the most reliable way for breast cancer to be detected at an early stage, where less radical treatment may be needed, and it is more likely that the cancer to be treated successfully with improved prognosis [2].

Many European countries have some form of screening for breast cancer, aiming to identify the early signs of cancer in asymptomatic women and lower the mortality rate [3]. To establish high-quality screening services, the European Commission Initiative on Breast Cancer (ECIBC) has published quality assurance guidelines, covering aspects such as the screening test, age range and screening intervals [4].

According to these guidelines, in the context of an organised screening programme, all asymptomatic women aged 50 to 69 are invited to attend breast screening every 2 years while the new European approach also suggests breast cancer screening with mammography for women from 45 to 74 years [5]. The interval between two rounds of screening is 2 years for most of the European countries [6]. Basic information of the population-based screening programmes in Europe is provided in the Supplemental Material [7, 8].

Quality assurance (QA) of image interpretation plays a key role in screening and diagnostic mammography, providing a framework for constant improvement of services offered to women [9–11]. Through continuous feedback breast radiologists can recognise and understand suboptimal clinical performance, determining potential image interpretation problems, and identifying training needs [12].

Although several quality assurance documents are now available and recommended by either European bodies, governmental departments, professional societies or national and regional organisations, the QA in screening and diagnostic mammography is quite heterogenous between countries and not always actively implemented across Europe [12].

The European guidelines for quality assurance in breast cancer screening and diagnosis also recommend that all radiologists involved in mammography have to participate in continuing medical education, internal and external audit procedures, formal appraisal, and performance benchmarking schemes as part of their QA activities [13]. The performance benchmarks include data such as recall rate, cancer detection rate, mean cancer size, cancer stage, sensitivity, specificity and positive predictive values from screening mammography, biopsy recommendation and biopsy performed [14]. The guidelines also outline some of the most important standards for maintaining or improving radiologists’ performance, including regular feedback on their performance, participation in training courses, multidisciplinary team meetings and reading a minimum number of mammograms per year [13].

In this context, the European Society of Breast Imaging (EUSOBI) conducted a survey among the members to collect information on radiologists’ preferences regarding QA measures of image interpretation in screening and diagnostic mammography.

## Materials and methods

### Survey design and distribution

The survey was designed by two board-certified radiologists, each with more than 10 years of experience in breast imaging, in collaboration with the PERFORMS team, an internationally well-known research group with extensive experience in assessing and improving the quality of clinicians’ performance in cancer screening. The aim of this survey was to assess the European QA status and breast radiologists’ preferences regarding QA measures of image interpretation in screening and diagnostic mammography.

The requirement for ethical approval was waived, as the study was classified as survey. Following approval by the experts of the EUSOBI executive board, the final questionnaire consisted of 25 questions that were split in two main sections. The first section requested anonymous personal information (such as age, gender, country of work, work setting and years of experience) as well as details about responders’ typical mammography workload. The second section covered questions relevant to quality assurance for mammography reporting in their country (see Supplemental Material). To ensure that the respondents would interpret the questions as intended, the survey was designed with structured questions in which they could select an answer from a set of multiple answers.

The survey was published online and an invitation to participate distributed to all EUSOBI members by the central EUSOBI office. The national breast radiology bodies across Europe were also contacted and invited to share the survey with their members. All responders were informed that participation was voluntary, and that confidentiality of their information would be ensured. The questionnaire was available online from April to July 2022. To maximise participation and increase the response rate, two email reminders were sent out during this period.

### Data analysis

The online software (Google Surveys) was used to collect the responses and spreadsheet data were exported for statistical analysis. Quantitative data was gathered using close-ended questions, while responses to open-ended questions were summarised and coded. Preliminary analysis of the data was undertaken using descriptive statistical tests, and the results were reported as frequencies and percentages to highlight important findings. The responses between subgroups were compared using the *χ*^2^ test. A linear regression analysis was conducted to investigate the relationship between variables. The Durbin-Watson (DW) statistic was used to detect the presence of autocorrelation in the residuals of a regression. Statistical calculations were performed using the IBM SPSS Statistics (version 28) statistical software (IBM SPSS Statistics for Windows, Version 28.0. Released 2021: IBM Corp.).

## Results

A total of 251 responders completed the online questionnaire. Females were the most responsive to the survey, representing 69.7% (175/251) of the survey population. The demographic characteristics are presented in Table 1.

**Table 1:** Demographic data of the survey responders

Demographic characteristic	*n*	%
Gender		
Female	175	69.7
Male	75	29.9
Prefer not to say	1	.4
Age		
< 30	1	.4
31–40	49	19.5
41–50	82	32.7
51–60	71	28.3
61–70	48	19.1
Years of experience		
< 3	13	5.2
4 to 8	48	19.1
9 to 14	42	16.7
15 to 20	47	18.7
20 to 25	34	13.5
> 25	67	26.7

Most responders were aged between 41 and 50 years (82/251, 32.7%), followed by the group of 51 to 60 years old (71/251, 28.3%), with the smaller proportions of responders being from 61 to 70 years (48/251, 19.1%) and less than or equal to 40 years old (50/251, 19.9%). Data from Table 1 indicate that following completion of training in radiology, consultant radiologists may not choose to undertake screening in their practice until later in their career. An estimated 26.7% (67/251) of responders reported to have more than 25 years of experience.

**Fig. 1:**
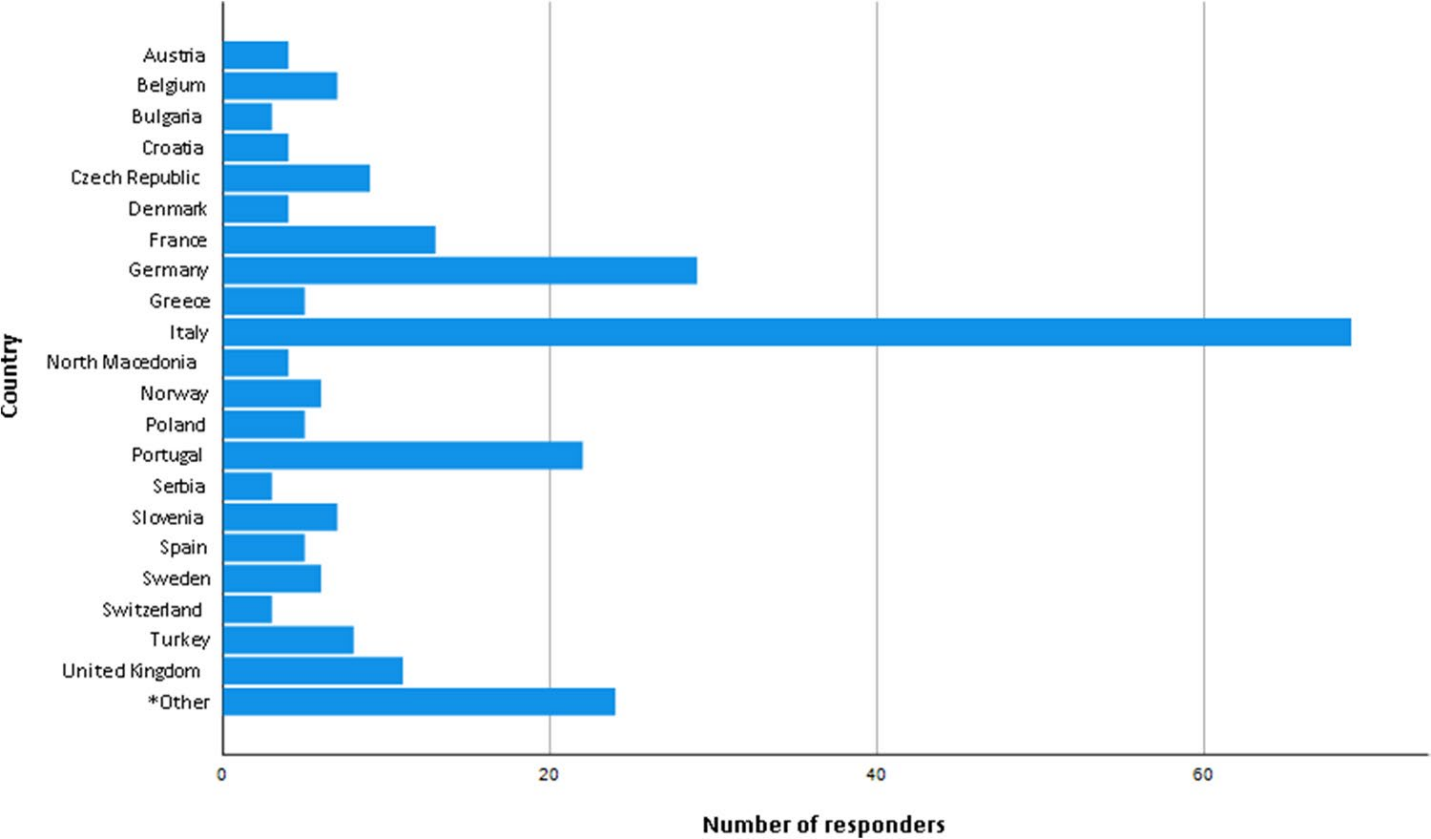
Countries where the responders were working at the time of the survey. *Other: countries in which less than three responders participated in the survey

In total, responses were received from 34 countries. Figure 1 presents countries where the responders were working at the time of the survey.

More than one-third of the responses (98/251, 39%) came from Italy and Germany. To account for potential bias introduced due to the high participation rate of these two countries, a linear regression test was carried out. Data screening did not indicate potential cause for concern regarding correlation of adjacent residuals (Durbin-Watson = 1.83). The regression equation produced a very small effect size (*R*^*2*^ = .016) indicating that country of work was not a significant predictor of the existence of established quality assurance measures in the workplace (F(1, 249) = 3.92, *p* = .05). There was no significant relationship between the country of work and the existence (or not) of established quality assurance measures (t(249 =1.98, *p* = .05).

### Workload in screening and diagnostic mammography

The majority of the breast imaging readers’ who participated in the survey were providing both breast screening and symptomatic services (137/251, 54.6%). In total, 85 responders (33.9%) were working in an academic hospital environment, 65 (25.9%) in a private setting (i.e., private hospital or private practice employed), 54 (21.5%) in a community hospital and the remaining 47 (18.7%) were employed in more than one setting.

Most of them devoted less than 50% of their time in screening (128/251, 51%) while the remainder were equal to or more than 50% (123/251, 49%). Most respondents reported to work within the national screening programmes (117/251, 46.6%), followed by those who work in opportunistic screening (88/251, 35.1%) or both (46/251, 18.3%).

The highest percentage of total participating respondents (109/251, 43.4%) read between 1000 and 4999 screening and diagnostic cases per year. However, the majority of respondents from Sweden and Poland read more than 20,000 cases per year and those from Greece, Czech Republic and UK read between 5000 and 9999 cases. Table 2 provides an overview of the number of cases read per year per country.

**Table 2:** Countries where the responders were based at the time of the survey and number of cases read per year

Country	Number of cases read per year						
	Less than 999 cases	1000 to 4999 cases	5000 to 9999 cases	10,000 to 14,999 cases	15,000 to 19,999 cases	More than 20,000 cases	Total
Austria	0	4 (100%)	0	0	0	0	4
Belgium	0	5 (71.4%)	1 (14.3%)	0	1 (14.3%)	0	7
Bulgaria	1 (33.3%)	1 (33.3%)	0	0	0	1 (33.3%)	3
Croatia	1 (25%)	2 (50%)	1 (25%)	0	0	0	4
Czech Republic	1 (11.1%)	1 (11.1%)	5 (55.6%)	1 (11.1%)	0	1 (11.1%)	9
Denmark	0	4 (100%)	0	0	0	0	4
France	3 (23.1%)	8 (61.5%)	2 (15.4%)	0	0	0	13
Germany	5 (17.2%)	12 (41.4%)	9 (31%)	2 (6.9%)	1 (3.5%)	0	29
Greece	0	2 (40%)	3 (60%)	0	0	0	5
Italy	14 (20.2%)	26 (37.7%)	21 (30.5%)	6 (8.7%)	2 (2.9%)	0	69
Norway	0	3 (50%)	2 (33.3%)	1 (16.7%)	0	0	6
Poland	0	1 (20%)	2 (40%)	0	0	2 (40%)	5
Portugal	5 (22.7%)	13 (59.2%)	2 (9.1%)	1 (4.5%)	1 (4.5%)	0	22
North Macedonia	0	3 (75%)	1 (25%)	0	0	0	4
Serbia	1 (25%)	2 (75%)	0	0	0	0	3
Slovenia	0	4 (57.1%)	3 (42.9%)	0	0	0	7
Spain	1 (20%)	2 (40%)	2 (40%)	0	0	0	5
Sweden	0	0	0	2 (33.3%)	0	4 (66.7%)	6
Switzerland	0	2 (66.7%)	1 (33.3%)	0	0	0	3
Turkey	0	4 (50%)	4 (50%)	0	0	0	8
UK	2 (18.2%)	2 (18.2%)	4 (36.4%)	3 (27.2%)	0	0	11
Other	3 (12.5%)	8 (33.3%)	5 (20.8%)	3 (12.5%)	4 (16.7%)	1 (4.2%)	24
Total	37	109	68	19	9	9	251

A chi-square test was used to confirm that there was a significant association between the country where the responders were based and the number of cases they read per year (*χ*^2^ (105, *N*=251) = 188.7, *p* <.001).

In addition, 33.1% (83/251) of respondents were working in a breast unit that diagnosed between 201 and 500 breast cancers per year compared to 29.9% (75/251) whose breast unit diagnosed between less than 50 and 100 breast cancers, 23.9% (60/251) between 101 and 200 cancers and 13.1% (33/251) more than 500 cancers in a year.

### Quality assurance of image interpretation in workplace

- ***Country guidelines on number of mammography cases read per year***

The majority of screening readers (84/251, 33.5%) reported that they should read more than 5000 cases annually to comply with their country’s guidelines, while for diagnostic readers, the most frequent answer (115/251, 45.8%) was that there are no guidelines on the number of cases a reader should examine per year. Table 3 demonstrates the breakdown of the responses by screening and diagnostic readers.

**Table 3:** Number of cases a mammography reader considers they should read annually to comply with their country’s guidelines

Screening mammography		Diagnostic mammography	
Number of cases	*N* (%)	Number of cases	*N* (%)
<1000 cases	17 (6.8%)	<100 cases	2 (0.8%)
1000–2999 cases	27 (10.8%)	100–299 cases	6 (2.4%)
3000–4999 cases	39 (15.5%)	300–499 cases	8 (3.2%)
>5000 cases	84 (33.5%)	>500 cases	71 (28.3%)
No guidelines	35 (13.9%)	No guidelines	115 (45.8%)
Don’t know	49 (19.5%)	Don’t know	49 (19.5%)

b. ***Quality assurance measures of breast imaging readers’ performance***

Over 18.7% (47/251) of responders considered that the number of mammograms read per year was the quality assurance measure of their breast imaging performance, followed by national benchmarking (46/251, 18.3%), personal benchmarking (36/251, 14.3%) and unit benchmarking (19/251, 7.6%). However, the majority (103/251, 41%) confirmed that all the aforementioned measures constitute the quality assurance for their performance.

Of the 133 responders (53%) who stated that they have established quality assurance measures in their workplace, 36 of them (27.1%) were working in a private setting and 97 (72.9%) in other settings, such as academic or community hospitals. The quality assurance measures included national benchmarking (14/133, 10.5%), number of mammograms read per year (20/133, 15%), unit benchmarking (23/133, 17.3%), personal benchmarking (24/133, 18%) or all the aforementioned measures (48/133, 36.1%). An estimated 3% (4/133) of responders reported other quality assurance measures, such as following the recommendations on the diagnosis and treatment of breast disease, produced by the European Society of Breast Cancer Specialists (EUSOMA). Of the 251 survey respondents, 198 (78.9%) reported attendance at Multidisciplinary Team (MDT) meetings on a regular basis.

For responders to ensure radiological-pathological correlation, they review the pathology reports and biopsy results (109/251, 43.4%), participate in team meetings (99/251, 39.4%), or explore other methods (43/251, 17.1%), such as consulting with a colleague.

c. ***Performance testing as part of breast imaging readers’ quality assurance***

As part of their QA, 71 responders (28.3%) had to participate in an either mandatory or voluntary performance test; 22 (31%) of them were working in a private setting. Although 71.7% (180/251) were not required to take a test as part of the QA, 77% (139/180) of them considered this would be beneficial. The main reasons given were to improve readers’ performance (62/139, 44.6%), service quality (62/139, 44.6%) and patient benefit (3/139, 2.2%). Other responses (12/139, 8.6%) included ensuring uniformity to certain practices, CPD (Continuing Professional Development) accreditation or continuous learning. Table 4 presents a breakdown of the responders who either participated in a performance testing or not based on the country of work.

**Table 4:** A breakdown of the responders who either participated in a performance testing or not based on the country of work

Country	Participation in performance test		
	Yes	No	Total
Austria	2 (50%)	2 (50%)	4
Belgium	4 (57.1%)	3 (42.9)	7
Bulgaria	1 (33.3%)	2 (66.7)	3
Croatia	1 (25%)	3 (75%)	4
Czech Republic	2 (22.2%)	7 (77.8%)	9
Denmark	0	4 (100%)	4
France	2 (15.4%)	11 (84.6%)	13
Germany	18 (62%)	11 (38%)	29
Greece	1 (20%)	4 (80%)	5
Italy	18 (26.1%)	51 (73.9%)	69
Norway	0	6 (100%)	6
Poland	0	5 (100%)	5
Portugal	0	22 (100%)	22
North Macedonia	1 (25%)	3 (75%)	4
Serbia	2 (66.7%)	1 (33.3)	3
Slovenia	0	7 (100%)	7
Spain	1 (20%)	4 (80%)	5
Sweden	1 (16.7%)	5 (83.3%)	6
Switzerland	1 (33.3%)	2 (66.7%)	3
Turkey	3 (37.5%)	5 (62.5%)	8
UK	9 (81.9%)	2 (18.1)	11
Other	4 (16.7%)	20 (83.3%)	24
Total	71	180	251

### Survey responders’ attitudes towards quality assurance of image interpretation

The majority of respondents (142/251, 56.6%) had the opinion that assessment test results do reflect their performance in the clinical practice, while 67.3% (169/251) considered that participation in regular testing would improve their performance.

**Fig. 2:**
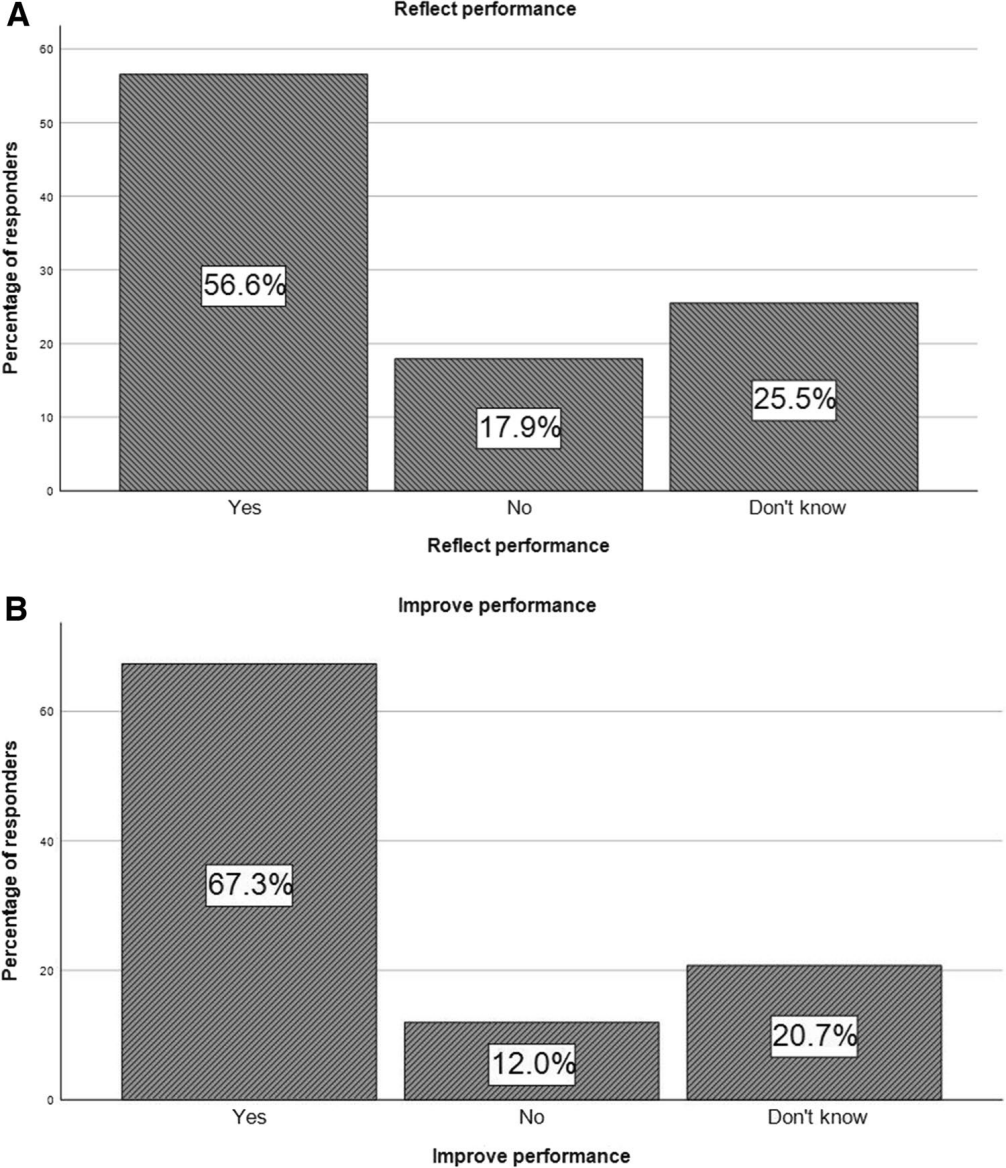
Regular performance testing plus (**A**) reflection on clinical practice and (**B**) performance improvement

Figure 2 demonstrates responders’ answers on testing plus (a) reflection on clinical practice and (b) performance improvement.

Over 65% (164/251) of responders felt that the most important way to improve their skills was by taking part in a performance test together with receiving feedback in their performance in real setting. Approximately 54.2% (136/251) of responders believed that a performance test should be compulsory, followed by 25.9% (65/251) who were not sure and 19.9% (50/251) who considered that such tests should not be mandated. However, when they were asked if they would be happy to take part in a mandatory performance test, the majority (190/251, 75.7%) agreed that a test set every 2 years (84/190, 44.3%) or once a year (72/190, 37.8%) would be beneficial. Approximately 15.3% (29/190) reported that readers’ participation in such test should be dependent on the number of mammograms read per year and 2.6% (5/190) only at the beginning of their career.

Respondents were also asked to describe any issues that could prevent implementation of performance tests in their settings. Overall, the most common barriers reported were lack of time and willingness from readers to be tested as well as lack of national/hospital funding to finance a performance test. Other reasons included difficulties in finding a suitable software that readers could use to perform the test and data protection issues that could arise from processing personal data and performance results.

## Discussion

This online survey was designed to assess the European QA status and breast radiologists’ preferences regarding QA measures of image interpretation in screening and diagnostic mammography. There were varied responses from 34 countries reflecting the difference between those countries with organised population screening programmes and those with opportunistic screening where QA of mammography image interpretation was less embedded.

Most screening readers reported that they should read more than 5000 cases per year in the screening setting in order to comply with their country’s guidelines. The European Commission Initiative on Breast Cancer (ECIBC) Guidelines Development Group suggested that mammography readers read between 3500 and 11,000 mammograms annually [4]. A recent study in the Norwegian screening programme demonstrated that there is a decreasing trend in radiologists’ sensitivity for annual reading volumes greater than 10,000 mammograms, a finding that could be related to their fatigue and time pressure at high workloads. Their analysis showed that optimal reader performance was achieved in radiologists reading between 4000 and 10,000 per annum [15].

In addition, one-third of the responders (those mainly from Bulgaria, Greece, Portugal, Serbia, Spain and Turkey) were either not aware of the breast cancer screening recommendations or did not have national guidelines in place. This finding remains of concern, as following screening recommendations is a crucial element for evidence-based management [16]. Such guidelines help to establish standards for the competence of personnel involved in reporting screening mammograms and promote continuous improvement in breast screening, offering high-quality services to women [17].

Similarly, most of the diagnostic readers reported that there were no guidelines on the number of cases a reader should examine per year. Although there are fewer symptomatic breast imaging guidelines, some countries have issued their own recommendations for diagnostic mammography. In the UK diagnostic readers are required to report on a minimum of 500 mammograms annually in symptomatic patients (patients who have signs or symptoms of breast cancer) [18]. The Danish clinical guidelines require diagnostic radiologists to evaluate at least 1000 mammography cases annually [19].

To address these differences while continuing to increase the standards of care, EUSOMA has developed a voluntary certification scheme for diagnostic and screening units, signifying that the quality standards have been achieved. According to this scheme, breast radiologists are required to interpret a minimum of 1000 mammography cases annually whereas those who participate in an organised population-based screening programme must have a workload of at least 5000 cases per year [20].

Compared to the European guidelines, the USA image readers are required to interpret a smaller number of cases. According to the American College of Radiology, the Mammography Quality Standards Act (MQSA) requires all personnel involved in reporting mammograms to examine at least 960 cases over a 24-month period [21].

More than half of the responders stated that they have established quality assurance measures in their workplace in order to work within a specialist framework. Although such measures are partially mandatory for private practice, the majority of responders working in a private sector are also required to follow QA practices. To comply with performance indicators and targets, all breast radiologists need to take part in internal and external audit procedures with corrective action being undertaken as required to achieve optimum performance and interpretation of breast images. Benchmarking provides the quantitative evidence to assess and monitor their radiological performance, determine potential problems and understand areas for improvement [22]. Recording and analysing individuals’ actual data is important although data often needs to be accumulated over several years to give robust cancer detection figures.

Performance feedback to radiologists is a quality assurance measure that facilitates awareness of their key performance indicators (such as recall rate, cancer detection, interval cancer rate and false-positive recall) but also highlights their need for further training [13]. Tailored to specific needs training activities and participation in continuing medical education improve readers’ image interpretation skills, reading performance and confidence in decision making — particularly to those who are less experienced [23]. It is evident that new or less experienced readers who regularly participate in assessment tests and receive feedback in their reading performance rapidly improve their cancer detection skills [24].

In order to maintain radiological performance and improve outcomes, it is also vital for readers to participate in regular MDT meetings with expert specialists from different disciplines. Attending such meetings is beneficial for feedback and educational purposes, as readers can discuss cases both pre- and post-operatively with other professional colleagues, access patients’ cytological and pathological results and better conform to evidence-based guidelines that outline the standards for readers and describe methods to best achieve them. However, there is limited evidence on the effectiveness of MDT participation to improve radiologists’ screening performance and patient outcomes [25].

According to European Guidelines for quality assurance in breast cancer screening and diagnosis, all breast screening personnel involved in reporting mammograms should participate in recognised external quality assessment schemes to reflect on their performance and take remedial action if needed, therefore improving patient safety [14]. Although most of the responders were not required to take part in a performance test as part of the QA, the majority of the answers indicated that a mandatory test every 2 years would be beneficial for skills appraisal purposes and service quality enhancement. Furthermore, though QA assessments could introduce bias as radiologists may interpret mammograms differently during a test set compared to real-life practice [26], the respondents believed that QA test results would reflect their performance in clinical practice. Recent evidence supported this statement, reporting a strong relationship between test set–based assessment schemes and real-life mammography reading performance [22].

There are limitations within the survey. Firstly, the questionnaire was kept short to encourage breast imaging readers’ participation (approximately 10 min). Consequently, it was not possible to add further questions regarding radiology quality assurance and standards (i.e., reasons for differences in QA implementation between countries, type of corrective action undertaken by radiologists). Females were the most responsive to the survey; however, the radiologists that participated were invited via their respective national breast bodies and therefore the total number of invitees cannot be verified. The age of the responders was fairly evenly distributed with the proportion (19.9%) of readers who were less than or equal to 40 years old reflecting the extensive years of postgraduate training required to become a specialised breast radiology consultant [6]. Although respondents were from 34 European countries, the most-represented countries were Italy and Germany (a linear regression test confirmed this did not affect the results) while countries such as Albania, Cyprus, Estonia, and the Netherlands had only one response. The findings, therefore, may not represent the preferences regarding QA measures of all breast imaging readers in Europe. A further survey could be of interest to analyse the points that were only raised within the current study.

## Conclusion

This is the first survey carried out that collects data on QA status in Europe and radiologists’ preferences regarding QA measures of image interpretation in screening and diagnostic mammography. The results demonstrate that although there are comprehensive and well-structured national and international guidelines in breast screening, the respective guidelines for readers involved in symptomatic breast imaging are not as well recognised and vary more substantially between different countries. However, in both cases, the vast majority of the breast imaging readers are aware of how important it is to have QA of mammography image interpretation in place as well as it being implemented for the services to be as effective.

### Supplementary information

ESM 1 (PDF 152 kb)

## Abbreviations

ECIBC: European Commission Initiative on Breast Cancer
EUSOBI: European Society of Breast Imaging
EUSOMA: European Society of Breast Cancer Specialists
MQSA: Mammography Quality Standards Act
QA: Quality assurance

## Declarations

### Guarantor

The scientific guarantor of this publication is Eleni Michalopoulou.

### Statistics and biometry

No complex statistical methods were necessary for this paper.

### Study subjects or cohorts overlap

N/A

### Methodology

- retrospective
- observational
- multicentre study
