# Supplementary material for: A survey by the European Society of Breast Imaging on radiologists’ preferences regarding quality assurance measures of image interpretation in screening and diagnostic mammography
Source: Eur Radiol. 2023 Jul 22;33(11):8103–11. doi: 10.1007/s00330-023-09973-7 (PMC10598074; doi:10.1007/s00330-023-09973-7)
Supplement: Supplementary file 1 — (PDF 152 kb) [file 330_2023_9973_MOESM1_ESM.pdf]

A survey by the European Society of Breast Imaging on radiologists' preferences regarding Quality Assurance measures of image interpretation in screening and diagnostic mammography

ELECTRONIC SUPPLEMENTARY MATERIAL

A. Supplementary Table: Basic characteristics of the breast screening programmes in Europe.

Country	Start	Double reading	Age screened (years)	Interval (years)
Austria	2014	Yes	45-69	2
Belgium	2001	Yes	50-69	2
Croatia	2006	Yes	50-69	2
Cyprus	2003	Yes	50-69	2
Denmark	2001	Yes	50-69	2
Estonia	2002	Yes	50-65	2
Finland	1987	Yes	50-69	2
France	1989	Yes	50-74	2
Germany	2002	Yes	50-69	2
Hungary	1995	Yes	45-65	2
Iceland	1987	Yes	40-69	2
Ireland	2000	Yes	50-64	2
Italy	1990	Yes	50-69	2
Luxemburg	1992	Yes	50-69	2
Malta	2007	Yes	50-60	3
Monaco	1994	Not available	50-80	2
Norway	1995	Yes	50-69	2
Poland	2006	Yes	50-69	2
Portugal	1990	Yes	45-69	2
San Marino	1993	Not available	35-74	2
Slovenia	2008	Yes	50-69	2
Spain	1990	Yes	45-69	2
Sweden	1989	Yes	40-74	2
Switzerland	1999	Yes	50-70	2
The Netherlands	1989	Yes	50-75	2
United Kingdom	1988	Yes	50-70	3

B. Preview of the online questionnaire

The 'Quality Assurance in Mammography across Europe' Questionnaire

You are invited to participate in our survey 'Quality Assurance in Mammography across Europe'. This short questionnaire asks for some anonymous personal information as well as details about your typical mammography workload, and quality assurance for mammography reporting in your country. While European Guidelines on acquisition and quality assurance in mammography are available, reporting habits and quality assurance in screening and

diagnostic mammography varies across Europe. The aim of this survey is to assess the European QA situation and radiologists' preferences regarding QA measures in screening and diagnostic mammography.

It takes approximately 10 minutes to complete the survey consisting of 25 questions. Your participation in this survey is completely voluntary. Your responses will be strictly confidential and data from this research will be reported only in the aggregate.

The survey is conducted by the European Society of Breast Imaging (EUSOBI) in collaboration with the PERFORMS team from University of Nottingham, UK.

Q1	What is your age?	< 30<br>31 - 40<br>41 - 50<br>51 - 60<br>61 – 70
Q2	What is your gender?	Female<br>Male<br>Non-binary<br>Prefer not to say
Q3	Which country do you work in?	
Q4	In which women's health area do you work?	a. Breast Screening<br>b. Symptomatic<br>c. Both Breast Screening & Symptomatic<br>d. High Risk Screening
Q5	In which setting do you work?	a. Academic hospital<br>b. Community hospital<br>c. Private hospital<br>d. Private practice employed<br>e. Private practice self employed
Q6	What percentage of your work is screening?	More than 50%<br>50%<br>Less than 50%
Q7	If you work in screening, is this within:	National screening programme<br>Opportunistic screening
Q8	Approximately how many years of experience do you have in reading mammographic cases?	< 3<br>4 to 8<br>9 to 14<br>15 to 20<br>20 to 25<br>>25
Q9	How many cases (2D, 3D and CE mammograms) would you estimate that you read in a year?	

Q10	How many breast cancers does your unit diagnose per year?	<50<br>51-100<br>101-200<br>201-500<br>>500<br>Don’t know
Q11	For screening readers:<br><br>According to your country’s guidelines, how many cases should a mammography reader read annually?	<1000 cases<br>1000-2999 cases<br>3000-4999 cases<br>>5000 cases<br>No guidelines<br>Don’t know
Q12	For diagnostic readers:<br><br>According to your country’s guidelines, how many cases should a mammography reader read annually?	<100 cases<br>100-299 cases<br>300-499 cases<br>>500 cases<br>No guidelines<br>Don’t know
Q13	What do you think are the quality assurance measures of breast imaging readers’ performance?	a. Personal benchmarking<br>b. Unit benchmarking<br>c. National benchmarking<br>d. Number of mammograms read per year<br>e. Other (text box)
Q14a	Do you have any established quality assurance measures of breast imaging readers performance in your workplace?	Yes<br>No
Q14b	If yes, what is it?	a. Personal benchmarking<br>b. Unit benchmarking<br>c. National benchmarking<br>d. Number of mammograms read per year<br>e. Other (text box)
Q15	How do you ensure radiological-pathological correlation?	
Q16	Are you attending tumor MDT meetings on a regular basis?	Yes<br>No
Q17	Is a reader’s performance testing part of your QA?	Yes<br>No
Q18	If it is not, would you like this to be implemented as part of your QA?	Yes<br>No
Q19	If you would like this to be implemented, why?	
Q20a	Do you see any issues to prevent implementation of such test?	No<br>Yes

Q20b		If yes, specify (text box)
Q21	Do you think it should be compulsory?	Yes<br>No<br>Don’t know
Q22a	Would you be happy to have a mandatory performance test?	Yes<br>No<br>Don’t know
Q22b	If yes, how frequently?	a. Once a year<br>b. Only at the beginning of your career<br>c. Accordingly with the number of mammograms read<br>d. Every two years
Q23	Do you think regular testing will improve your performance?	Yes<br>No<br>Don’t know
Q24	Do you think test results reflect your performance in the clinical practice?	Yes<br>No<br>Don’t know
Q25	What do you think is more important to improve your skills?	a. A performance test<br>b. Feedback on your performance in real setting<br>c. Both
